# Mechanism and Performance Analysis of Nanoparticle-Polymer Fluid for Enhanced Oil Recovery: A Review

**DOI:** 10.3390/molecules28114331

**Published:** 2023-05-25

**Authors:** Yuanxiu Sun, Weijie Zhang, Jie Li, Ruifang Han, Chenghui Lu

**Affiliations:** 1 College of Petroleum Engineering, Liaoning Petrochemical University, Fushun 113001, China; 2 Baikouquan Oil Production Plant of Petrochina Xinjiang Oilfield Branch, Karamay 834000, China; lj99@petrochina.com.cn (J.L.); bkqhrf@petrochina.com.cn (R.H.); lchui@petrochina.com.cn (C.L.)

**Keywords:** nanoparticle-polymer fluid, polymer, nanoparticles, EOR, IFT, wettability

## Abstract

With the increasing energy demand, oil is still an important fuel source worldwide. The chemical flooding process is used in petroleum engineering to increase the recovery of residual oil. As a promising enhanced oil-recovery technology, polymer flooding still faces some challenges in achieving this goal. The stability of a polymer solution is easily affected by the harsh reservoir conditions of high temperature and high salt, and the influence of the external environment such as high salinity, high valence cations, pH value, temperature and its own structure is highlighted. This article also involves the introduction of commonly used nanoparticles, whose unique properties are used to improve the performance of polymers under harsh conditions. The mechanism of nanoparticle improvement on polymer properties is discussed, that is, how the interaction between them improves the viscosity, shear stability, heat-resistance and salt-tolerant performance of the polymer. Nanoparticle-polymer fluids exhibit properties that they cannot exhibit by themselves. The positive effects of nanoparticle-polymer fluids on reducing interfacial tension and improving the wettability of reservoir rock in tertiary oil recovery are introduced, and the stability of nanoparticle-polymer fluid is described. While analyzing and evaluating the research on nanoparticle-polymer fluid, indicating the obstacles and challenges that still exist at this stage, future research work on nanoparticle-polymer fluid is proposed.

## 1. Introduction

Global demand for oil and gas is expected to grow at an annual rate of 2–3% in the coming decades [1,2]. This phenomenon is more prominent in commercial and personal transportation activities, and this demand is directly related to economic conditions. At the same time, petroleum fuels have been the main source of energy. Petroleum fuels have the advantage of high-energy density compared with other alternative energy sources [3,4]. As the demand for energy increases, a continuous supply of energy becomes particularly necessary. Nevertheless, the petroleum reserves of conventional fields are dwindling. In 2008, the global economic crisis erupted due to the continued decline in oil exploitation of existing fields and the declining interest in discovering new oil reserves [5,6]. Therefore, enhanced oil recovery has also become an issue of great concern and importance to the oil industry and even to the industrial sector. Nevertheless, traditional oil recovery methods cannot give full play to the potential of reservoir development, which will leave approximately 50% or more of the original reserves [7,8]. In summary, it is necessary to adopt certain technologies in the petroleum industry to enable the extraction of more and more oil reservoirs. In particular, the development of new technologies or materials will be crucial for extracting crude oil in the future.

Tertiary recovery is a recovery method used to enhance oil recovery. Its widespread promotion and focused application have led to a significant increase in crude oil recovery as well as total crude oil production, which has given an important impetus to the stable development of the oil industry. Tertiary oil recovery is a technology for re-exploitation of remaining oil by chemical, physical, biological and other methods on the basis of primary and secondary oil recovery, which is called EOR [9]. The injected chemicals improve the properties of the oil, which is the key to extracting residual oil from the narrow pores of the reservoir [10]. These traditional tertiary recovery technologies can be divided into chemical flooding, thermal flooding, miscible flooding, and microbial flooding [11,12]. Thermal EOR refers to injecting thermal fluid into the oil reservoir or the oil reservoir is burned to form a moving thermal fluid. It is a method of using heat energy to reduce the viscosity of crude oil and increase its flow capacity. Chemical flooding has shown promising potential to overcome these challenges and is one of the successful techniques for enhanced oil recovery. The main measures include surfactant flooding, polymer flooding, nanofluid flooding, and composite system flooding [13,14,15]. The polymer has high viscoelasticity because it has a long-chain structure with high molecular weight. It stretches oil drops and oil films during the flow process, thereby increasing its carrying capacity. Polymer flooding can obtain a better mobility ratio, reduce the relative permeability of water, improve the water phase sweep volume, and thus improve recovery efficiency [16,17,18]. Polymer degradation and surfactant adsorption limit the use of chemicals in harsh reservoir conditions, and chemical agents also have the disadvantage of being environmentally unfriendly and uneconomical [19]. Due to the aforementioned limitations on enhanced recovery, the field is being upgraded to the next level by introducing new methods or materials.

In response to the aforementioned challenges, nanoparticles have attracted a lot of attention from researchers. Nanomaterials and their technologies have been applied in different fields, such as machinery manufacturing, electronic information, chemical medicine, aerospace, food safety, transportation, environmental protection, oil and gas, and other fields [20,21,22,23]. Over the past few decades, nanomaterial-enhanced oil recovery (N-EOR) technologies have been remarkably successful against the limitations of traditional oil displacement techniques [24]. Nanomaterials can be classified into nanoparticles, nanometer clay, and nanometer emulsion based on the external shape and internal structure of the material. Nanoparticles are divided into metal oxide nanoparticles, organic nanoparticles, and inorganic nanoparticles [25]. According to relevant reports, the nanoparticles commonly used in N-EOR include SiO_2_, TiO_2_, MoS_2_, Al_2_O_3_, ZnO, etc. [26,27,28,29]. The reason for this attention is that the nanofluids can reduce the IFT between oil and water, changing the wettability of surfaces between a rock and crude oil, and structural separation pressure is generated in the three-phase contact zone and reduces oil viscosity [30,31,32,33,34]. The polymers are mainly divided into two categories, namely biopolymers and synthetic polymers. The former includes natural polymer materials such as xanthan gum and Guar gum, while the latter includes polymers such as HAM and polyethylene. As a chemical oil displacement agent, high molecular weight partially hydrolyzed polyacrylamide (HPAM) has been widely used in the field of tertiary oil recovery, which plays an important role in steady production and increased production of oil fields. However, under the influence of complex environments such as high temperatures and high salt, the polymer is prone to bacterial degradation and flexible shrinkage. In addition, it is also easy for a precipitation phenomenon in small pore sizes to occur under the influence of cations and its absorptive properties. This study shows that the addition of nanoparticles (metallic nanoparticles, non-metallic nanoparticles) can improve the performance defects of polymers. It shows better heat resistance, salt resistance, and chemical resistance than traditional polymer fluids. The main representative materials are SiO_2_ and HPAM, and the hydrogen bond formed by the ion interaction between SiO_2_ and the HPAM surface increases the dispersibility of the nanoparticle-polymer solution. The quantum size effect of nanoparticles increases the fluidity of nanoparticle-polymer fluid in the narrow formation. The unique physical and chemical properties of the nanoparticles increase the viscosity of the polymer nanofluid and prevent its degradation and precipitation behavior. The IFT, wettability, and stability of the nanoparticle-polymer are discussed in this paper. Nanoparticles placed between polymer chains can increase the internal friction of the solution, resulting in improved fluidity associated with it. Nanoparticle-polymer fluid has good interfacial activity, and the addition of nanoparticles reduces the interfacial tension between oil and water. The nanoparticle-polymer fluid changes the wettability of the system from oil-wet to water-wet or medium-wet, displacing the residual oil from the reservoir pores. The stability of polymer nanofluids is described. While analyzing and evaluating the research on nanoparticle-polymer fluid, indicating the obstacles and challenges that still exist at this stage, future research work on nanoparticle-polymer fluid is proposed.

## 2. Polymer Flooding Mechanism and Influencing Factors

Polymer flooding is a technology in which a polymer solution of a “water-soluble polymer” is used as the displacing agent. Adding appropriate water into the polymer solution adjusts the water solubility of the molecular polymer, and the fluidity of the water is reduced by increasing its viscosity. The crude oil comes out of the pore channel preferentially, and the strata water is retained in the primary pore channel as much as possible during the process of polymer flooding, thus improving oil recovery [35,36]. At the macroscopic level, it mainly depends on increasing the viscosity of the displacing fluid and reducing the mobility ratio between the displacing fluid and the displaced fluid, so as to expand the swept volume [37]. The polymer produces a stretching effect on the oil film or oil droplet during flow because of its inherent viscoelasticity, which increases the carrying capacity and thus the microscopic oil displacement efficiency is improved [38]. The mechanism of polymer flooding technology is to mix water and polymer to raise the viscosity of the water to the desired value. The decrease in the mobility ratio is realized and the viscosity of the water is improved, so that the areal sweep coefficient is improved [39]. The entanglement of polymer molecular chains in the pores increases the flow resistance of the water in the highly permeable parts, which also contributes to the improvement in sweep efficiency [40]. The hydrophilic groups in the polymer chain play a leading role. The ‘water sheath‘ is formed outside the polymer molecule under the action of the hydrophilic groups, and internal friction is generated between the two. The hydrophilic group decomposes after entering the aqueous solution to produce a chain element. These links carry electrodes and the links repel each other under the action of the electrodes, causing the molecular chain of the polymer to be stretched in water and become more adhesive [41]. Polymers are viscoelastic and have pseudoplastic and shear-thickening behavior when subjected to shear stress while passing through porous media. The remaining oil is displaced by stretching, stripping, and shear thickening of the polymer [42]. Polymer molecules undergo deformation after flowing through the pore, and the polymer solution at the blind end pore points to the directional force of the connected pore channel, which is perpendicular to the direction of flow. Based on the above action, the polymer solution enters the blind end to displace oil [43]. The flow direction of the polymer in the pore tends to develop more towards the end of pores and the deep retention area of pore throat than that of the viscoelastic fluid, which is beneficial to displace crude oil in deep narrow pores.

### 2.1. Common Polymers in Oil Displacement

The polymers commonly used in chemical flooding are mainly divided into two main categories, natural polymers and synthetic polymers [44]. Natural polymers are generally referred to as biopolymers and are usually derived from natural plant products or organisms, in other words, they are the polymers derived from cells or substances outside of cells [45]. At present, biopolymers are widely used in various industries, including the petroleum industry. Polymers such as xanthan gum, guar gum and modified cellulose are typical examples of polymer flooding used to enhance oil recovery. The most common biopolymer used as an oil repellent in the petroleum industry is xanthan gum. The main reason for this is that xanthan gum has multiple advanced structures in solution. The side chains in the molecule are reverse bound around the backbone to form a helical structure, and then a three-dimensional double helical network structure is formed depending on weak forces. Based on the above-mentioned reticulated three-dimensional structure, xanthan gum has a very strong ability to withstand acid, alkali, salt and temperature [46]. It does not lose its properties and retains about 80% of its original viscosity under high salt and high temperature conditions [47]. In addition, it also has good water solubility, thickening and other performance characteristics. Guar gum is a natural non-ionic polysaccharide, and is one of the best-known natural polymers in terms of water solubility. Its solubility allows it to have excellent dispersion in cold or hot water without agglomeration. Low concentration guar gum produces a stable aqueous solution with high viscosity at low shear rates. As the shear rate increases, the polymer exhibits shear thinning behavior [48]. The most important feature of the guar gum molecule is that its structure is very similar to that of xanthan gum, and this similarity gives it a strong affinity for cellulose [49]. The network structure of cellulose determines it has good properties of high mechanical shear resistance and high temperature resistance [50]. Cellulose is often surface modified to meet the needs of polymer flooding technology, and the main reason is that its specific molecular structure tends to produce uneven dissolution or dissolution problems [51]. In addition to the above polymers, biopolymers such as scleroglucan, shell polyose, schizophyllan, Welan gum and other polymers have also been used in polymer flooding oil recovery technology.

Synthetic polymers are usually classified as polyacrylamide (PAM), hydrolyzed polyacrylamide (HPAM) and hydrophobic associative polyacrylamide (HAPAM). PAM is a water-soluble linear polymer, which is formed by the free radical of acrylamide or other derivatives, and appears as an agglomerate in aqueous solution [52]. The active amide group and anionic carboxyl group on its molecular bond will undergo physical and chemical reactions with a variety of substances [53]. PAM has the functions of thickening, flocculation, resistance reduction and purification, and it contains high molecular carbon chains, so it has biological resistance. The molecular chains of PAM curl into clumps, resulting in insufficient extension of the active groups in the chain. Therefore, in practical application, it is generally required to be hydrolyzed in advance, and its performance depends on its degree of hydrolysis. HPAM is a copolymer of PAM and sodium polyacrylate or is a product containing a carboxyl group that is produced by hydrolysis of the amide group [54]. HPAM is a kind of straight chain polymer, and the structure of the molecule is a flexible chain structure with random curling, which avoids the disadvantage of PAM curling into clumps. HAPAM is a polymeric derivative formed by introducing a copolymerized monomer into the polymer backbone. The charged ionic groups cause the macromolecular chain of polymers to produce intramolecular or intermolecular physical association under the action of electrostatics, hydrogen bonds and van der Waals force. Thus, a large space network structure is formed, that is, a dynamic physical crosslinking network structure [55]. In the process of oil displacement, groups are mainly introduced by water-soluble polymer molecules, and oil displacement is achieved by hydrophobic action groups. This is because the association of polymer molecular chains increases the area of flow dynamics, whereby the intermolecular association mainly depends on the intermolecular force [56]. In addition, other types of polymers such as crosslinked polymers, amphiphilic polymers, heat-resistant and salt-tolerant polymers and core-shell polymers are also used in enhanced oil recovery technologies for tertiary oil recovery [57]. It was found that polymer flooding with HPAM or XG as a displacement agent has been widely applied in petroleum production and has achieved remarkable results [58,59].

### 2.2. Influence of Salinity

From Section 2.1, we can learn that biopolymers are mostly stable under conditions of high salinity and high temperature. However, biological and synthetic polymers are susceptible to degradation. The degradation of biopolymers is more severe compared to synthetic polymers. The cell debris from the degradation of xanthan gum polymers can accumulate in pores, causing pore blockage resulting from formation damage. In addition, biopolymers also have the disadvantage of being oxidized by external conditions [60,61]. The influencing factors of viscosity of the polymer aqueous solution mainly include: relative molecular mass of polymers, degree of hydrolysis, mass concentration, salinity, high valence cations, pH value and temperature, etc. It shows that the viscosity of a polymer is affected by both its own factors and environmental factors [54]. HPAM is widely considered to be the most commonly used polymer for oilfield applications. This section describes the influencing factors of polyacrylamide and its derivatives in the process of oil displacement.

**Figure 1:**
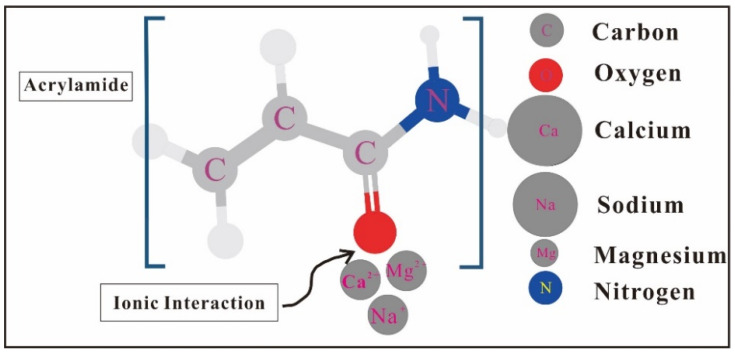
Ion-dipole interactions between cations and C=O bonds [64].

**Figure 2:**
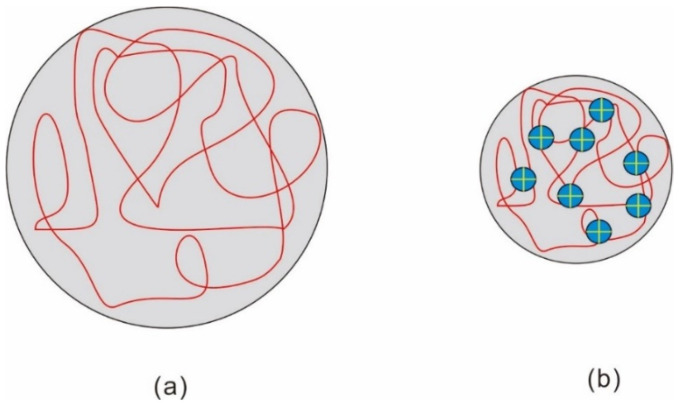
Linear shrinkage of HPAM flexible chain (**a**) HPAM polyelectrolyte solution (**b**) HPAM polyelectrolyte solution combined with cations.

Based on the analysis of the molecular structure of HPAM, the molecular structure is flexible chain and the molecular state is random coil. The multiple charges are distributed on the molecular chain of HPAM making HPAM a polyelectrolyte solution [62]. When low concentration salt is added to HPAM, the molecular chain is greatly extended, showing high viscosity. The strength of solution ions increases with the increase in salt concentration, and the low molecular opposite ions penetrate the macromolecule opposite ions. This behavior shields the effective charge and causes the polymer chain to shrink, resulting in a rapid reduction in viscosity [63]. When CaCl_2_ and MgCl_2_ are added, the viscosity of HPAM solution decreases sharply with the increase in salt concentration. This is because with the increase in high valence ions, the hydrophobic association is electrostatically shielded by the counter charge, and the molecular chain curls. The structure of the association network in the solution is destroyed, which reduces the viscosity of the solution. With the increase in divalent cation mass concentration, the divalent cation has stronger ion-dipole interaction with the amide group, which weakens the bond strength of N-H and C=O; the ionic bond reaction is shown in Figure 1 [64]. The above reasons reduce the electrostatic repulsion on the polymer chain and reduce the hydrodynamic radius of HPAM macromolecules, and the macromolecular coil shrinks, resulting in a gradual decrease in the viscosity of the polymer solution [65,66]. Yang et al. also confirmed this view in the performance experiment of crosslinked polyacrylamide microspheres [67]. Figure 2 shows the structural changes of the HPAM flexible chain. This is because as the salt concentration increases, the electric double layer and hydrolysis layer on the HPAM molecular chain are thinned by the externally strengthened electrolyte, and the repulsion of the charge on the macromolecule main chain of the macromolecule is weakened, causing the molecular chain to curl and the size to be small. Al^3+^ is prone to intramolecular crosslinking with COO- on the same polymer molecule, forming a large number of linked polymer coils [68].

### 2.3. Influence of Temperature

Temperature is an external interference factor that must be considered because it can accelerate the degree of polymer hydrolysis and enhance the interaction between the polymer molecular chains and cations in solution. The solution properties, rheology and stability of the polymer system change significantly, and eventually phase separation occurs. PAM has the characteristics of high solute molecular weight, a long molecular chain, and curling into a network structure in water, resulting in a high sensitivity to environmental temperature [69]. Wang et al. measured the infrared transmission spectra of PAM solutions with different concentrations by using a spectrum experiment device.

The results of the infrared band experiment show that when the temperature rises above 40 °C, the heating causes the PAM solution to be evaporated, resulting in small bubbles in the PAM solution. The spectral peaks of the PAM solution produce significant changes, indicating that the temperature has an effect on the structure of the PAM solution [70]. According to the molecular structure and flocculation mechanism of PAM, if the dissolution temperature is too high, the molecular chain of the polymer is easy to break, and then the bridging action of the polymer is destroyed thus reducing the effect of its use [71]. The PAM solution is hydrolyzed under the action of temperature. The amide groups produced by high temperature hydrolysis produce strong ion-dipole interaction with cations, thus C=O and N-H chemical bond strength is weakened [72]. The amide group of HPAM is further hydrolyzed into a carboxylate group in a high-temperature environment. The increase in negative charge caused by this behavior causes the polymer to precipitate under the action of cations, and the polymer skeleton cannot remain stable [73]. The occurrence of precipitation was confirmed by the experiments of Abidin et al., who found that HPAM dissociated at high temperatures and the polymer would precipitate in the presence of Ca^2+^ and Mg^2+^ [19]. If precipitation does not occur, the interaction between cations and anions will also cause the viscosity of HPAM to decrease. Wang et al. investigated the effect of temperature on the viscosity and shear rate of ultra-high molecular weight salt-resistant polyacrylamide. The experimental results show that the higher the temperature, the lower the viscosity of the solution. Under the same shear conditions, with the increase in temperature, the molecular chain gradually shrinks and the shear rate decreases. It is proved that the molecular chain of polyacrylamide in water has the property of expanding with lower temperature [74].

### 2.4. PH

PH is an important factor in determining the properties of polymer solutions. The carboxyl group on the main chain polymerization is sensitive to PH, and the degree of reaction of the polymer depends on the degree of reaction between the H^+^, OH^−^, and -COOH groups. The phenomenon of acidification will occur when the polymer solution is in a low pH environment. When the H^+^ concentration is higher, the negative charge on the polymer chain combines with hydrogen ions. When the environment is alkaline, the concentration of OH^−^ is higher and the electrostatic repulsion between ions increases [75]. Sheng et al. proposed that the addition of alkali made the viscosity of the PAM solution higher than the initial value, while the viscosity of a xanthan gum system decreased slightly [76]. However, Luo et al. believed that the addition of NaOH reduced the viscosity of polymer solutions, and the viscosity of the binary combinational system was lower than that of the pure polymer solution. However, with the increase in hydrolysis time and the concentration of alkali and polymer, the viscosity of the solution will gradually increase. When the alkali content exceeds the critical value, the cation will have a shielding effect on the polymer solution [77]. The addition of an alkali can promote the hydrolysis of an amide group on the polymer chain, which increases the amount of negative charge on molecular chain and increases the electrostatic repulsion between molecules and within molecules. The electrostatic repulsion causes the polymer molecular chains to change from a curly state to an uncoiled state, which increases the viscosity of the polymer. Chen et al. made a further study on the effect of alkali on the molecular-aggregation morphology of HPAM. They believe that the alkali can promote the hydrolysis of PAM with low degree of hydrolysis, and the repulsive force between carboxylic acid groups makes the molecule stretch and the solution viscosity increases. Conversely, continuing to add alkali will reduce the viscosity of the polymer solution.

### 2.5. Self-Factors of Polymer

The flow of a polymer solution is determined by the properties of polymer and reservoir pores, and the adsorption and retention polymer in formation pores will lead to the decrease in porous media permeability and even blockage [78]. The high viscosity and adsorption properties of the polymer cause the retention of the polymer in the formation. The greater the viscosity of the polymer solution, the greater the degree of entanglement between the polymer molecular chains, which will drive the migration of clay particles, resulting in the blockage of narrow pores [79]. In addition, the hydrodynamic diameter of the polymer increases with the increase in the polymer solution viscosity, which accelerates the formation of a network structure and causes the retention of the polymer in small pore throats [80]. The retention of the polymer in the formation will reduce the pore throat size of the reservoir, narrow the seepage channel, and even completely block the channel.

The network structure formed by the association of hydrophobic associative polymer leads to the retention of the polymer at the pore throat, which increases the adsorption of polymer molecules and increases the adsorption capacity [81]. When the polymer comes into contact with the reservoir rock, the molecular structure of the polymer tends to be adsorbed on the rock surface, resulting in a polymer adsorption phenomenon. In addition, polymer fluidity in the reservoir is affected by the retention rate of the polymer and the pore volume of the reservoir rock.

## 3. Nanoparticles Commonly Used in Oil Displacement

At present, the commonly used nanomaterials can be divided into inorganic nanomaterials, organic nanomaterials and composite nanomaterials. With the in-depth study of preparation methods, nanomaterials have been widely used in different industries (such as medicine, optoelectronics, oil fields, etc.), and gradually developed from experimental research to industrialization. The most common nanomaterials used in tertiary oil recovery applications fall into three categories: nanoparticles, nano-clays, and nano-emulsions [82]. The commonly used nanoparticles include non-metallic oxides (SiO_2_), metal oxides (TiO_2_, Al_2_O_3_, NiO_2_, ZrO_2_, Fe_2_O_3_, etc.) and organic nanoparticles (nanotubes, graphene, nanocellulose crystals, etc.) [83]. Common nanoparticles with potential significance for enhanced oil recovery are briefly discussed.

### 3.1. Nanoparticles of Metal Oxide

Metal oxide nanomaterials exhibit physical and chemical properties such as high specific surface area, electron mobility, thermal stability, mechanical strength and surface defects because of their unique nano-size. The wide application of metal-oxide nanomaterials in petroleum engineering has attracted the wide attention of researchers. Karimi et al. synthesized ZrO_2_ nanoparticles by a sol-gel method, and the carbonate rocks aged in crude oil were placed in nanofluids for aging experiments. They found that the carbonate slabs changed from oil-wet to water-wet after the aging time exceeded 48 h [84]. The researchers found that the wettability of rock can be effectively improved by the synergistic action between Al_2_O_3_ nanoparticles and cetyltrimethylammonium bromide (CTAB) [29]. It was found that replacing different types of nanoparticles had the same good effect, e.g., the nanofluid containing Fe_3_O_4_. The nanofluid can carry the surfactant CTAB to effectively improve rock wettability, and the oil displacement experiment found that the nanofluid can make the recovery rate reach 60% [85]. Zargar et al. grafted TiO_2_ nanoparticles onto the surface of quartz, and the nanofluid reduced the interfacial tension from 36.4 mN/m to 2.6 mN/m. They suggest that nanoparticles adsorb at the oil-water interface to form a layered structure, thus reducing the oil-water interfacial tension [86]. The combination between the carboxyl group of oleic acid and the hydroxyl group on the surface of nano TiO_2_ is chemically bonded, and the surface of OA-TiO_2_ has both hydrophilic and lipophilic properties [87].

### 3.2. Non-Metallic Oxide Nanoparticles

The surface of SiO_2_ has abundant hydroxyl functional groups, and there are three kinds of hydroxyl groups with different bonding states [88]. Therefore, it is easy to carry out secondary modification or chemical modifications, which has caused a high research interest in petroleum engineering, especially in the field of enhanced oil recovery. Nazari et al. tested the effects of different types of nanoparticles on the wettability of reservoir rocks and found that SiO_2_ had the best effect on changing the wettability of oil-wet rocks [89].

SiO_2_ is prone to agglomeration due to the interaction of SiO_2_ surface groups and size effects, and its actual action volume is usually much larger than the nanoscale. The dispersion of nanoparticles in a polymer solution is difficult due to the repulsion between nanoparticles and polymer organic molecules, which results in uneven properties, inconsistent interface energy and decreased rheological properties of the solution [90]. Therefore, researchers use different methods and modifiers to modify the surface of SiO_2_ or compounds with polymer. The modification methods of SiO_2_ mainly include surface chemical modification and inorganic coating. Nguyen et al. introduced modified SiO_2_ nanoparticles into core-shell coating polymers and constructed a new system with different proportions of surfactants. The results show that the new composite system can reduce IFT and increase viscosity at the critical concentration, and has high heat and salt resistance [91]. The presentation of the types of nanoparticles in this section is not limited to the above content. The application of more types of nanoparticles will be more fully reflected in the following sections.

## 4. Nanoparticle Reinforced Polymer

**Figure 3:**
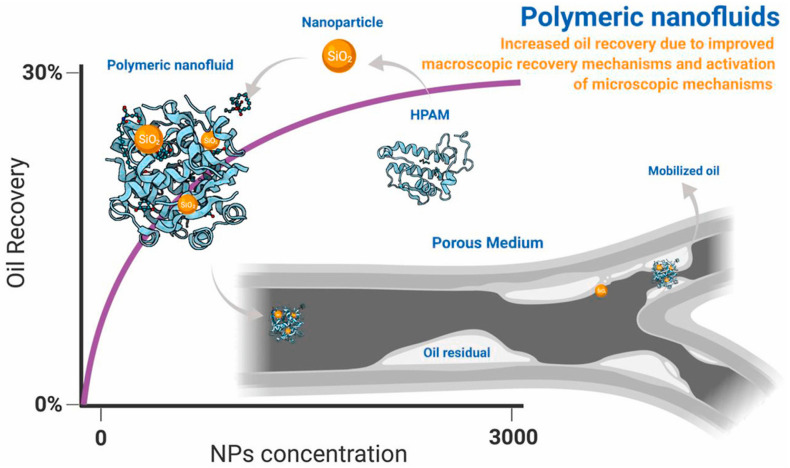
Macro-micro theoretical mechanism of SiO_2_ nanoparticles-polymer flooding system [95].

Polymer flooding is a tertiary oil recovery method, which is one of the most widely used tertiary oil recovery methods [92]. However, polymers are limited by relative molecular mass and molecular structure. This method cannot adapt to complex reservoirs such as high temperature, high salt, low porosity and low permeability because of its poor comprehensive performance, which affects the improvement of oil recovery [93]. Nanoparticles have a series of favorable characteristics such as small size, high surface and interface activity, easy chemical modification and thermal stability [94]. The nanoparticle reinforced polymer is created by introducing nanoparticles into the polymer, such as silica, nano alumina, titanium oxide, nickel oxide, etc. Nanoparticles are bonded to polymer molecules by hydrogen bonding or other intermolecular forces, and the stability of the nanoparticle-polymer composite structure is enhanced. Combined with the characteristics of nanoparticles, the viscosity, temperature resistance, salt resistance and shear resistance of polymers can be improved. Therefore, nanoparticle-polymer fluid is a kind of oil displacement system with broad application prospects. Figure 3 shows the displacement diagram of the nanoparticle-polymer fluid during oil displacement [95]. Table 1 summarizes the effects of polymer and nanoparticle-polymer on IFT, contact angle and oil recovery.

### 4.1. Quantum Size Effect and Chemical Bonding between Nanoparticles and Polymers

**Figure 4:**
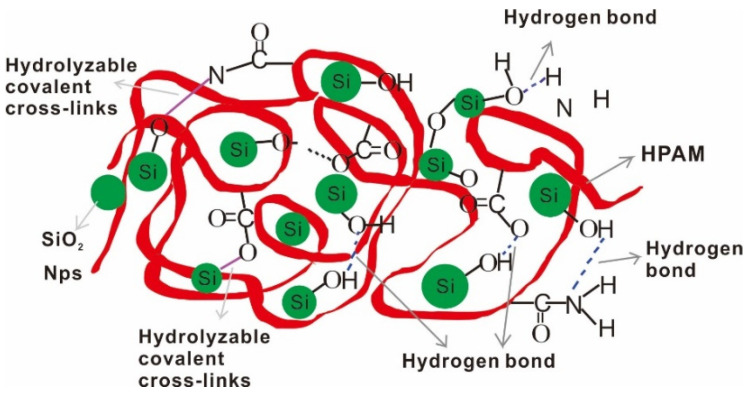
Potential interactions between SiO_2_ and HPAM [104].

Nanoparticles improve the properties of polymer solutions and enhance the strength and toughness of materials because the size of the nanoparticles is similar to the size of polymer molecules. The surface activity of nanoparticles makes it easy for them to bond with the polymer backbone and the single atom on the surface reacts with the polymer molecular chain [101]. Therefore, the high interfacial energy between the nanoparticles and the polymer can connect the two and improve the elastoplasticity of the solution when the external stress changes. Some researchers have studied the chemical bonding between nanoparticles and polymers by taking silica and PAM as an example. When Mandal et al. prepared PAM/silica nanocomposites, they found that the addition of silica reduced the particle size of polymer composites. The interaction between polymer chains and silica particles was characterized by Fourier-transform infrared spectroscopy, and the results show that the strong interaction between the polymer chain and silica is due to the bonds that are formed between the hydrogen bonds of NH_2_ and the oxygen bonds of Si-O [102]. There is a strong interaction between silica nanoparticles and polymers with carbonyl groups due to the formation of hydrogen bonds, which causes polymer macromolecules to adsorb onto the surface of the silica nanoparticles and acts as a physical crosslinking agent to connect different polymer chains [103]. Figure 4 shows the interaction between SiO_2_ and HPAM [104].

The above work only introduces the chemical reactions between nanoparticles and polymers and does not consider the effects of cations on chemical bonding in the presence of salt. The cations bind to the negative charge on the polymer chain, and the oxygen atoms on the tetrahedral structure of silica can form ion-dipole interaction with cations to the greatest extent. Cations are attracted by silica and polymer in competition with each other, reducing the degree of influence of cations on the polymer molecular chain [72]. The cations in the salt solution react with the oxygen atoms on the surface of the nanoparticles, shielding the attack of cations on polymers. The bonding between the hydroxyl group of nanoparticles and the amide group of polymers is stronger, and the spread range of the nanoparticles is wide [105].

GO is an oxide of graphene, which has other properties such as mechanical properties, chemical stability and thermal conductivity [106]. Oxygen-containing functional groups such as hydroxyl, epoxy and carboxyl groups exist on the surface of GO, which provides sufficient reaction sites for surface chemical modification and has been extensively studied in enhanced oil recovery [107]. Aliabadian et al. synthesized two different types of graphene oxide nanosheets (S-GO and E-GO) to investigate the functionalization effects between graphene oxide nanosheets and HPAM aqueous dispersions. It was found that the interaction between polymers and graphene is mainly through hydrogen bonding, electrostatic attraction and hydrophobic interaction. Among these, the hydrogen bonds are produced by the ionic reaction of the -OH group on GO with the carboxyl and amide groups on the main chain of HPAM, and the hydrophobic interaction is generated by the non-polar chains of the HPAM molecule reacting with the non-polar sheet of GO [108]. The strength of the interaction between nanoparticles and polymers is significantly enhanced by a variety of interactions. Regarding the formation of hydrogen bonds between GO and graphene, the researchers found three types of hydrogen bonds between GO and HPAM: the hydrogen bond between the carboxyl group of HPAM and the hydroxyl group of GO, the hydrogen bonds formed by -NH_2_ of HPAM and -COOH in GO and the hydrogen bond between -NH^3+^ and the epoxide and carboxyl groups in GO. The existence of the hydrogen bond greatly improves the viscosity and thermal stability of HPAM [109].

### 4.2. Improvement of Polymer Properties by Nanoparticles

#### 4.2.1. Improvement of Viscosity

The increase in viscosity is the main reason for the decrease in the oil-water mobility ratio, which is achieved by increasing the concentration of the polymer in the aqueous solution. However, increasing polymer concentrations has its limitations in terms of oil displacement. The main reason is that there are many large micelles in the high concentration polymer solution, which reduces the solubility of the polymer in water. Meanwhile, salinity and high temperature have adverse effects on rheological properties of the HPAM solution. The high pressure in the formation will cause shear stress on the polymer, causing the polymer chain to break, and the chemical reactions of the polymers are accelerated by the high temperatures, causing the molecular chains to shrink. This phenomenon can be addressed by placing nanoparticles between polymer chains to increase the viscosity of the fluid.

**Figure 5:**
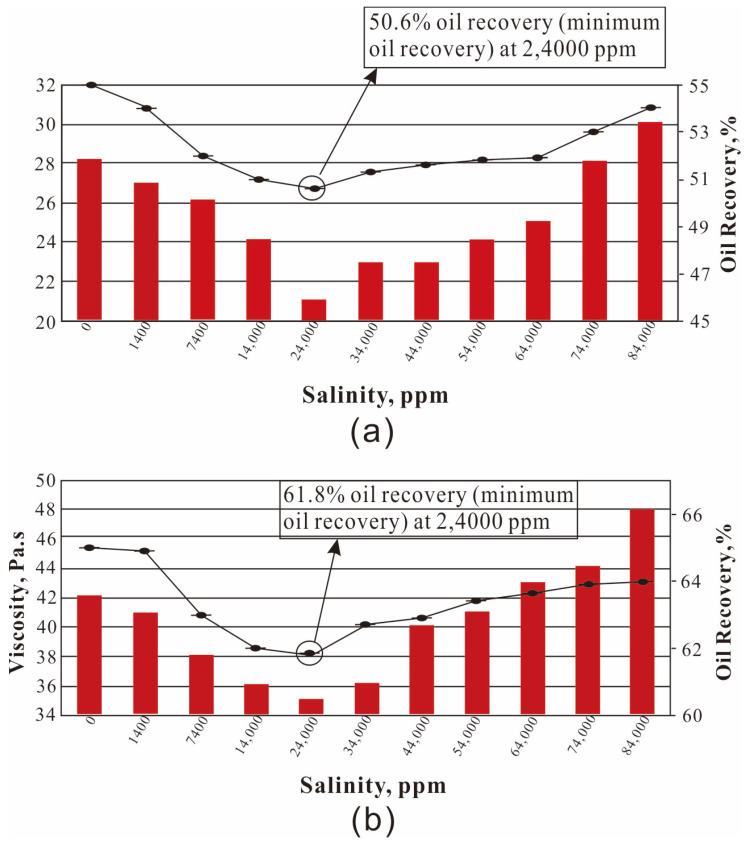
(**a**) 1000 ppm polymer solution (**b**) 1000 ppm nanoparticle-polymer solution [72].

**Figure 6:**
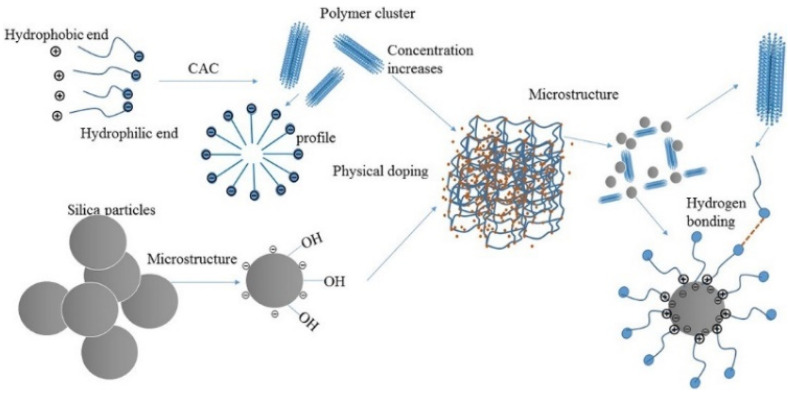
Mechanism of increasing fluid viscosity by combining silica nanoparticles with amphiphilic polymers [112].

It is mentioned in Section 4.1 that the surface groups of nanoparticles have certain characteristic activity and can be used as crosslinking agents. Therefore, the rheological property of the displacing fluid is improved, and the viscosity of the displacing fluid is increased by the nanoparticles, thus the sweep efficiency is improved. Maghzi et al. evaluated the effect of SiO_2_ on the rheological properties of polyacrylamide solutions, and found that the addition of silica nanoparticles can increase the viscosity of polymer solutions. This is because the nanoparticles form an interpenetrating network structure with the polymer chain, which further increases the molecular volume of the polymer, resulting in an increase in solution viscosity. In addition, it was found that the nanoparticles improve the pseudoplastic behavior of polymer solutions at low shear rates [110]. They further investigated the effect of SiO_2_ nanoparticles on polyacrylamide in the presence of salt. The recovery rate of the SiO_2_-PAM solution was lower than that of the pure polyacrylamide solution. We can see from Figure 5 that the 1000 ppm nanoparticle polymer solution has higher viscosity and recovery than the polymer solution [72]. The decrease in polymer adsorption and the increase in solution viscosity were caused by silica nanoparticles, and the idea was confirmed in a numerical simulation by Khalilinezhad [111]. The mechanism by which silicon dioxide increases the polymer solution is shown in Figure 6 [112]. The spatial network structure is formed under the action of hydrophobic and hydrophilic groups when the concentration of the amphiphilic polymer reaches a certain concentration. However, the structure of polymers aggregated in this way is unstable. With the addition of silica, the free macromolecular polymers are connected by silica under the combined action of electrostatic and hydrogen bonding, which enhances the strength of the connections between polymers and enhances the loose structure of the polymer molecules. In the study by Zhu et al. they found that under 20 °C conditions, the apparent viscosity of 2.0% HAPAM is 75 mPa·s, and after the addition of 1.5% SiO_2_, the apparent viscosity of HAPAM increases to 150 mPa·s. This is mainly due to the dense network structure formed between HAPAM/silica [113].

This property is not only reflected in the SiO_2_-PAM solution, but also has the same effect on other nanoparticles and polymers. The viscosity of the polymer can be uniformly increased when the hydrophilic fumed silica acts on the sulfonated polyacrylamide solution [95]. In Cheraghian’s study, they found that the addition of TiO_2_ nanoparticles increased the viscosity of the polyacrylamide solution. The results show that at zero shear rate, the viscosity of the solution increases by about 47% after adding 2.3 wt% TiO_2_ nanoparticles [114]. The amide group existing in each polymer chain results in the polymer chain being adsorbed or grafted onto the silica surface, which is the main reason for the increase in viscosity. The interconnect function of silica particles leads to the formation of complex macromolecular structures in the solution, which increases the hydrodynamic radius and increases the viscosity of the polymer solution. In addition to the role of nanoparticles as crosslinking agents, hydrogen bonding, electrostatic attraction and hydrophobic interactions also improve the strength of the interaction between nanoparticles and polymers. The formation progress of complex spatial network structures is accelerated and eventually shows higher viscosity.

#### 4.2.2. Stability of Shear Property

The addition of nanoparticles can not only increase the viscosity of the system, but also improve the pseudoplastic behavior of the polymer solution and enhance the stability of the system. Zhu et al. studied the effects of SiO_2_ on shear resistance and rheological behavior at high temperature and salinity. They observed that the shear viscosity of the nanoparticle-polymer fluid increased with the addition of SiO_2_ nanoparticles, and they show better shear resistance than the corresponding PAM solution [113]. Rezaei et al. induced HPAM molecules with clay nanosheets to investigate the rheological properties of nanoparticle-polymer fluid at typical shear rates. The results show that the addition of nano-clay particles improves the pseudoplasticity and shear thinning property of polymer solutions at typical shear rates [99]. It also has good shear stability for metal oxide nanoparticles. For example, Al_2_O_3_ nanoparticles also showed better stable shear performance at different electrolyte concentrations and temperatures [5]. The size, morphology and surface chemical structure of nanoparticles can remain stable after shearing because of the good shear resistance. In addition, there is electrostatic and hydrogen bonding between nanoparticles and polymers, and the molecular network structure can be partially recovered after shear failure.

#### 4.2.3. Thermal Stability and Salt Tolerance

The limitations of polymers under external conditions such as temperature and salinity have been described in the second part of this paper. In view of the problem that polymers are prone to precipitation and degradation under high temperature and a high salt environment, researchers began to try to add nanoparticles to improve the thermal stability and salt tolerance of polymers. The performance of the SiO_2_/HPAM/NaCl system at 60 °C was studied by Chen et al. It was found that the viscosity of the HPAM solution decreases significantly with the increase in temperature when SiO_2_ is not added and the trend of viscosity reduction is reduced after the addition of SiO_2_, which indicates that the addition of SiO_2_ enhances the temperature resistance of the HPAM solution. The polymer will be hydrolyzed to produce more carboxyl groups at higher temperatures. Therefore, the effect of modified nanoparticles on polymer properties has been widely studied in recent years. The effect of nanoparticles on polymer properties is uncertain because the surface modification groups of nanoparticles are different [115]. Cao et al. prepared amphiphilic nano-silica with an amino function by modifying the amino groups and octyl groups of silica. The comparison of the polymer, SiO_2_-polymer and modified SiO_2_-polymer showed that the modified SiO_2_-polymer showed a better viscosity effect under the influence of salinity. The results indicated that the amine-functionalized SiO_2_ nanoparticles significantly improved the temperature tolerance and salt tolerance of HPAM [116]. Jia Han et al. obtained amphiphilic SiO_2_ nanoparticles by attaching an amino group to one side of the surface of nanoparticles and an alkyl group on the other side. The unmodified hydrophilic SiO_2_ nanoparticles and amphiphilic SiO_2_ nanoparticles were added to an HPAM aqueous solution and heated to 75 °C for shear test. The viscosity retention rate of amphiphilic SiO_2_ nanoparticles for the HPAM solution is higher than that of unmodified hydrophilic SiO_2_ nanoparticles system at high temperature. In addition to the above nanoparticles, other nanoparticles have also enhanced the temperature and salt resistance of polymers. Examples include graphene oxide, Janus-SiO_2_ and other nanoparticles [109,117,118].

The electrostatic interaction between nanoparticles and polymers is stronger than the hydrogen bond and van der Waals forces between polymer molecules. Therefore, the network structure formed by the polymer and nanoparticles is less affected by temperature, that is, the nanoparticle-polymer fluid has better temperature resistance. The inorganic/organic composite structure formed by nanoparticles and polymer molecules is beneficial to inhibit the curling of polymer molecules under high concentrations of salt ions, which improves the rigidity of polymer molecules and helps to form a network structure between molecules. Nanoparticles can still interact with the polymer molecules to enhance the strength of the molecular network structure under the condition of a high concentration of salt ions. Therefore, a polymer modified by nanoparticles has better salt resistance.

### 4.3. Advantages of Nano Polymer Fluids

**Figure 7:**
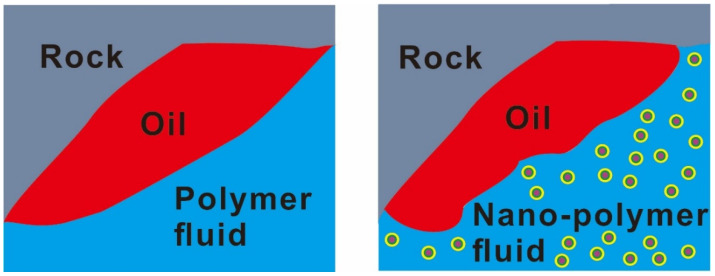
Comparison of the properties of ordinary polymer fluid and nanoparticle-polymer fluid.

The injected nanoparticles are coupled with the polymer, which has a synergistic effect on improving oil-recovery efficiency. Nanoparticle-polymer fluid flooding systems can yield higher oil and gas production than surfactant, polymer or nanoparticle systems. Figure 7 shows a comparison of the properties of nanoparticle-polymers and conventional polymers during oil displacement. Meanwhile, the losses during oil displacement can be reduced when appropriate nanoparticles and polymers are selected. Yousefvanda et al. conducted three sets of comparative experiments; water flooding, polymer flooding and nanoparticle-polymer flooding, respectively, in the presence of salt. The results show that the nanoparticle-polymer fluid has a better oil displacement effect than the other two solutions [96]. The reason for the good oil displacement effect is that the addition of nanoparticles increases the viscosity of the nanoparticle polymer system and it can change the oil wettability of the rock to water wettability or partial water wettability to the greatest extent. It was found that the oil displacement effect of the nanoparticle-polymer fluid was better than that of a surfactant-polymer system and alkali-surfactant-polymer system [108].

Therefore, we can conclude that the introduction of nanoparticles into polymers can effectively improve polymer mobility. The viscosity-increasing ability and shear resistance of a nanoparticle-polymer are improved in different degrees compared with that of the single polymer, and the possibility of improving its pseudoplastic behavior is increased. This means that nanoparticle-polymer fluid has a stronger priority in improving viscosity, which can improve the displacement efficiency of crude oil to a greater extent. The interaction between polymers and nanoparticles has a synergistic effect, which can improve the rheological properties of polymers and the stability of nanoparticles. The applicability of the polymer in the reservoir and the sweep efficiency of the displacing fluid can be improved with the addition of nanoparticles to polymer solutions, and the large drag coefficient and residual drag coefficient are established in porous media, thus improving the displacement efficiency of the tertiary recovery process.

## 5. EOR Mechanism of Nanoparticle-Polymer Fluid

**Figure 8:**
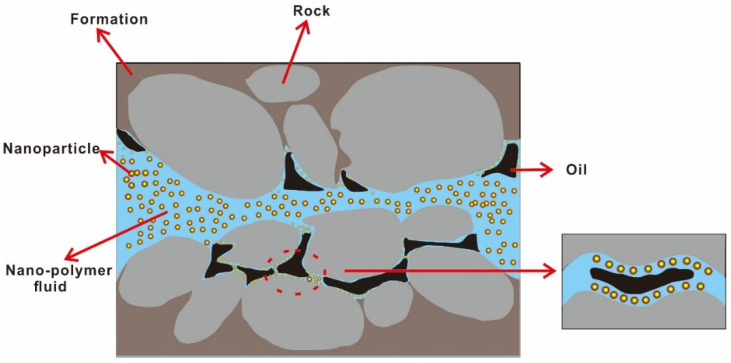
EOR mechanisms of nanoparticle-polymer within the porous media.

The hydrolysis and degradation of the polymer can be effectively inhibited by introducing nanoparticles into the polymer, and it is conducive to the formation of a dense network structure. The existing recovery-enhancement methods of chemical flooding, gas flooding and thermal recovery have limitations in technology, economy or the environment. The nanoparticle-polymer fluid can effectively expand the swept volume, improve oil displacement efficiency, reduce remaining oil saturation and improve crude oil recovery when it is used as a displacement agent. The mechanism of enhanced oil recovery is mainly to reduce the interfacial tension of oil and water, change of wettability, stability, etc., which is helpful for the nano polymer fluid to displace residual oil in porous media. The displacement mechanism of nanoparticle-polymer in pores is shown in Figure 8 and Table 2 summarizes the positive effects of nanoparticle-polymer fluid on enhanced oil recovery.

### 5.1. IFT

Interfacial tension refers to the surface free energy between two immiscible liquids, which determines the distribution of fluids in the formation. Reducing IFT is one of the ways to enhance oil recovery. This is because the distribution and flow of crude oil in the reservoir are affected by capillary forces. The oil droplets in the rock can have a good flow when the capillary number reaches a certain value, and the capillary force depends on the interfacial tension between the fluid phases [132,133]. Interfacial tension is very important for reducing the capillary force and increasing the capillary number, and it is one of the key parameters for enhancing oil recovery [134]. Ultra-low interfacial tension can greatly increase the capillary number and reduce the binding strength of the rock to the crude oil when the IFT is reduced to 10^−3^ mN/m, and the flow velocity of the remaining oil in the reservoir is significantly increased [135]. The relationship between capillarity force and interfacial tension parameters is shown in the following equation [136]:

$$P_{C}=\frac{2\sigma \cos\theta }{r}$$

Here, σ is fluid-oil IFT, θ is the angle between the wetting fluid and the solid surface, r is the effective radius of the interface, and $P_{C}$ is the capillary pressure. Polymeric microspheres and nanospheres can swell when meeting with water and then reduce water permeability due to their ability to reduce the capillary force and change the water flow path. Thus, the displacement efficiency is improved [137]. Nanoparticle-polymer fluid has excellent interfacial activity. It can be adsorbed at the oil-water interface to reduce interfacial tension, increase the capillary number and reduce the driving pressure of crude oil, which is beneficial to the flow of crude oil in the reservoir and further improves oil recovery. Therefore, the reduction in interfacial tension at the micro level is of great importance to improve oil-drive efficiency.

**Figure 9:**
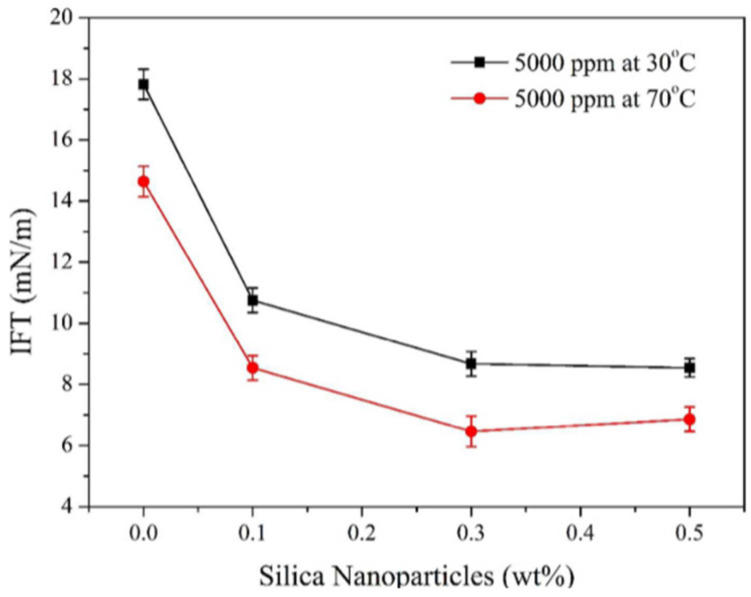
Interfacial tension as a function of nanoparticle concentration and experimental temperature [6].

Nanoparticles have strong interfacial adsorption, which helps to reduce IFT between oil and water. This is because nanoparticles such as Al_2_O_3_, MgO, SiO_2_ and TiO_2_ have good IFT reduction capabilities [138,139]. Combined with the properties of nanoparticles, the interface-reduction properties of nanoparticle-polymer fluids have been widely discussed by researchers. In 2012, the oil-water (xylene-water) interfacial-tension behavior of polymer fluids grafted with nanoparticles was investigated by Alvarez et al. The experimental data show that the concentration of nanoparticles has a non-negligible importance to the reduction in interfacial tension, that is, the higher the concentration, the smaller the oil-water interface [119]. The main reason is that both grafted nanoparticles and polyacrylamide are hydrophilic. Water promotes the migration and adsorption of the interface, and moderate hydrophilicity reduces the interfacial tension. The oil displacement schemes of polymers (P), surfactant-polymers (SP), nanoparticle-polymers (NP) and nano-surfactant-polymers (NSP) were compared. It was found that both NP and NSP solution systems had a better effect on reducing the interfacial tension when the concentration of nanoparticles was 1.0 wt%, and the minimum value was lower than the corresponding P/SP fluid in each case [97]. Based on the above studies, Qi et al. measured the effect of a polymer-coated silica solution on the interfacial tension of heavy oil and water. The water contact angle decreased significantly from 76° to 62 with a concentration of 1.0 wt% nanoparticle-polymer fluid, and the oil-water interfacial tension decreased from 27 mN/m to 14 mN/m. This indicates that the solution has interfacial activity and can reduce oil-water interfacial tension at neutral pH [120]. Biopolymers are more affected by temperature than synthetic polymers. Therefore, the behavior of the xanthan gum-silica system to reduce the interfacial tension of oil and water was evaluated when the temperature was considered. As shown in Figure 9, it was found that the IFT of the system is not only related to the concentration of added nanoparticles, but also to the temperature. The IFT of the system decreased from 17.8 mN/m to 10.75 mN/m at 1.0 wt% nanoparticles and 30 °C. A similar trend exists at 70 °C, where the IFT decreased from 14.64 mN/m to 8.54 mN/m [6]. The reduction in oil-water interfacial tension is due to the high adsorption, suspension stability and strong interaction of nanoparticles. The decrease in IFT value is conducive to the flow of residual oil in the pore and increases the movement of oil droplets in the pore throat.

**Figure 10:**
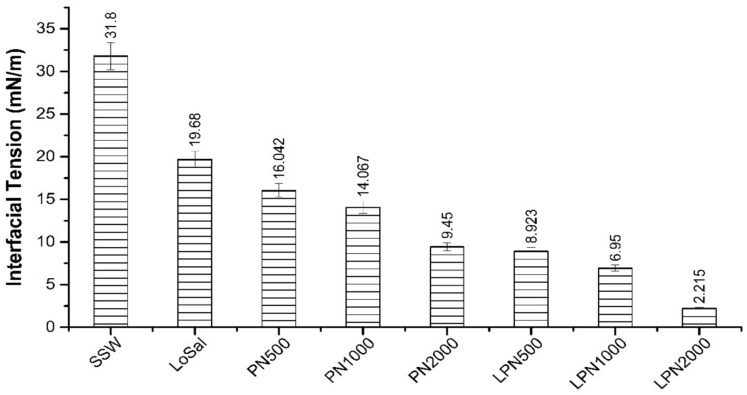
Schematic diagram of changes in the effects of salinity and concentration on interfacial tension [1].

The anionic polymer-citrate coated Fe_3_O_4_ nanoparticle suspension was developed by Izadi et al., and the interfacial tension between oil and water was measured at high temperature and high salinity. The interfacial tension of the crude oil system was 11.23 mN/m when water was injected, while the interfacial tension of the nanoparticle-polymer fluid was reduced to 7.92 mN/m. It is concluded that a polymer coated Fe_3_O_4_ solution can meet the requirements of harsh salinity and high temperature through the presentation of experimental data. The interfacial tension of the oil-water system can be reduced to a certain extent, and the interfacial tension is sensitive to the increase in the concentration of nanofluids [4]. In addition, they found that interfacial tension was closely related to the concentration of the nanoparticle-polymer fluid, and they are inversely proportional to each other within a certain range. Nanomaterials consisting of various types of nanoparticles produce layered structures at the interface between oil and fluid, which acts as an intermediary and results in a reduction in interfacial tension between immiscible fluids. This is because individual nanoparticles of CuO/TiO_2_ exhibit a collaborative ability to reduce IFT, and the nanoparticle-polymer fluid produces better results than their individual effects with the addition of PAM [2]. Xanthan gum-coated ZnO/SiO_2_ nanocomposites were prepared by Ali et al., and the interfacial tension between oil and water with different salinities was measured. As shown in Figure 10, the initial IFT value of the oil-low salinity system was 19.68 mN/m, and the IFT was reduced to 9.45 mN/m by adding the appropriate concentration of nanoparticle-polymer fluid. It can also be concluded from the figure that increasing the concentration of the nanoparticle-polymer fluid and reducing the salinity are conducive to the reduction in interfacial tension [1]. The reason for the decrease in interfacial tension is consistent with Bahraminejad ‘s view, that is, a layered structure is formed at the interface between crude oil and nanoparticle-polymer fluid, so that IFT is reduced.

Nanoparticles have many unique properties and interfacial behaviors compared to conventional polymer flooding. For example, nanoparticles and polymers can form a mixed layer at the interface between a fluid and crude oil, which is beneficial to the reduction in oil-water interfacial tension. From the above data, it can be seen that oil recovery is improved to a corresponding extent after the addition of nanoparticles compared with a single polymer. The results show that the nanoparticle-polymer fluid has excellent interfacial activity, which can reduce the interfacial tension between high-viscosity crude oil and water, and increase crude oil production. However, the influence of external factors such as salinity and temperature should be taken into account in the experiment of interfacial tension.

### 5.2. Wettability

**Figure 11:**
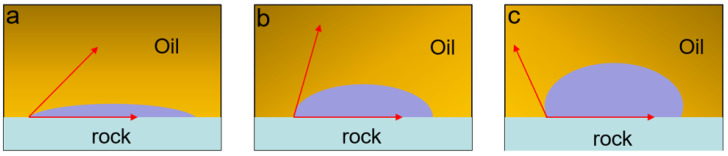
Relation between contact angle and wettability (**a**) water-wet (**b**) medium-wet (**c**) oil-wet.

Wettability, like interfacial tension, is also one of the important parameters for enhanced oil recovery. It plays a key role in reservoir evaluation and dynamic analysis. It reflects the microscopic distribution of residual oil and original fluid in the reservoir. Wettability is defined as “the tendency of one fluid to spread on or adhere to a solid surface in the presence of other immiscible fluids” [140]. The reservoir rocks may exhibit different wettability, which may be oil-wet, water-wet, or medium-wet, and this wetting behavior depends on the interaction between the rock and the crude oil. The contact angle is a key index to measure wettability. The wettability shows absolute hydrophilia when the contact angle is 0°; it is water-wet when the contact angle is between 0° and 60°; it is medium-wet when the contact angle is between 60° and 90°; it is oil-wet when the contact angle is between 90° and 180°; it shows absolute lipophilia when the contact angle is 180°, as shown in Figure 11.

Most reservoir rocks have mixed-wet wettability due to the crude oil environment in which the rocks exist. The crude oil on the oil-wet rock surface adheres firmly to the rock surface, resulting in the formation of an oil film. As a result, it takes more energy to peel the oil film off the rock surface [141]. The capillary force on the contact surface of oil-water is more inclined to the displacement phase due to the surface characteristics of the rock. The capillary force produces resistance to the flow of crude oil, resulting in the reduction in the displacement efficiency of residual oil. Therefore, the wettability of the rock surface changes from oil-wet to water-wet or oil-wet to neutral wet, which is beneficial to recovery efficiency [142]. The reason why it is difficult to displace residual oil in oil-water porous media is that it adsorbs on the rock surface in the form of a continuous oil film. During polymer flooding, a stronger force is produced during the flow of polymer solutions, which is conducive to the peeling of the oil film, and then improves the displacement efficiency of residual oil. However, although the polymer can alter wettability, this change is not significant and is not sufficient to create a very strong water-wet condition. In response to this problem, nanoparticles have been further studied. These can effectively diffuse to the solid surface, thereby altering the wettability of the rock surface. Table 3 summarizes the contact angle range and chemical bond characteristics of nanoparticles with different properties.

**Figure 12:**
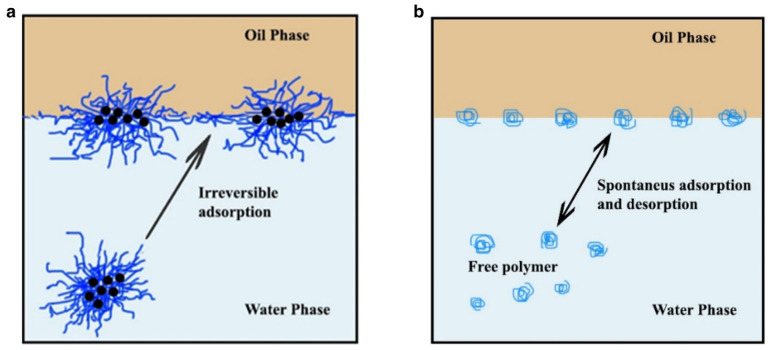
(**a**) Irreversible adsorption of PAM-grafted SiO_2_ nanoparticle (**b**) dynamic adsorption of traditional polymer [144].

The wettability of a solid surface relates directly to the solid-fluid and fluid-fluid interactions. The wettability of solid surfaces can be changed by introducing nanoparticle-polymer fluids into the solid-liquid system. Joonaki et al. used propanol as a dispersing agent for nanoparticles, and the change of contact angle was measured after adding Fe_2_O_3_, Al_2_O_3_ and SiO_2_ nanoparticles. The wettability of rock changes from neutral wettability to water wettability with the increase in solution concentrations. The results showed that SiO_2_ was the most effective in changing the wettability, which changed the contact angle from 134° to 82° [143]. Studies have shown that replacing propanol with PAM also has excellent results. Sandstone cores with different properties were tested for wettability and a qualitative assessment of fluid wettability changes in the nanoparticle-polymer was established. They found that SiO_2_-HPAM and Al_2_O_3_-HPAM reduced the contact angle from 111.8 to 31.0° and 25.1°, respectively, through experimental measurements of Al_2_O_3_ and SiO_2_ at a different solution concentrations and reservoir temperatures. The wettability of the sandstone core is converted to a strong water-wet state because of the irreversible adsorption of nanoparticles to the rock surface [5]. Figure 12 shows the comparison of adsorbability between a polymer-grafted nanoparticle solution and a polymer solution [144]. At the same time, the nanoparticles also have the effect of dispersion, and their combined action changes the wettability of rocks [145].

**Figure 13:**
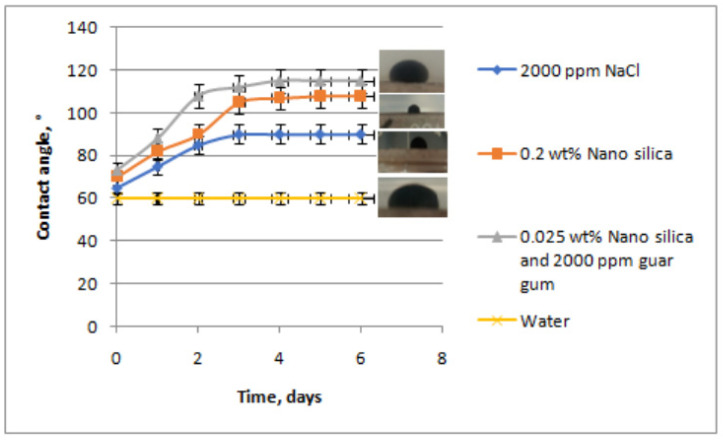
Variation curves of contact angle with time for different fluids and corresponding photos of oil drops [100].

Bila’s research shows that polymer-coated silica nanoparticles can alter the wettability of oil and rock, and the contact angle decreases from 143.30° to 48.75° with the addition of silica nanoparticles [121]. The interaction between ionic bonds produces new surface roughness leading to the interface energy between rock and water being lowered and so the oil film on the surface is destroyed. In addition, the contact area of nanoparticles is large, thus forming a wet surface. Figure 13 shows the curves of contact angle with time for the four tested solutions and the corresponding photographs of the oil drops. Analysis of the data shows that a silica and guar gum polymer solution can effectively convert surfaces from lipophilic to hydrophilic. Silica nanoparticles are easily adsorbed in sandstone when guar gum and silica are mixed as a displacing fluid, which is very active in influencing the contact angle and helps to improve wettability [100].

Nanomaterials have shown a good effect in changing the wettability of reservoir rocks. Nanomaterials can enter channels in porous media and change their surface characteristics. Moreover, nanomaterials are easily adsorbed on the surface of the rock to form a hydrophilic layer. This is because a strong hydrogen bond is formed between the nanoparticles and the solution, increasing the surface free energy. This phenomenon changes the wettability of the system from oil-wet to water-wet or medium-wet, releasing the remaining trapped oil from the pore of the reservoir rock. The change in rock wettability not only contributes to the displacement of oil from the small pores of reservoir rock but also increases the relative permeability of the oil and improves the recovery rate.

### 5.3. Stability Analysis

It is important to evaluate the stability of a nanoparticle-polymer fluid before using it in oil displacement. The type, size, shape and additive quantity of nanoparticles will affect the stability of nanofluids. The nanoparticles have high collision probability and easy aggregation for the nanoparticle-polymer fluid with a high volume fraction, which makes the stability of the nanoparticle-polymer fluid decrease. The settlement of agglomerates is caused by the interaction of different forces between particles [146]. Pore throats may be blocked and rock properties altered when nanoparticles form aggregates [147]. Krishnan et al. studied the convective heat transfer of nanofluids with spherical and flaky MgO, respectively. It was found that the surface tension of the flake MgO nanofluid was higher, which indicated that it had a stronger van der Waals force and faster aggregation of nanoparticles [148] The classical framework theory used to describe the stability of colloids in suspension is the DLVO theory. The theory mainly addresses the balance between two opposing forces, namely van der Waals (VDW) attraction and electrostatic repulsion. Increasing the repulsive force between particles can improve the even distribution of particles in the solution, which prevents the particles from precipitating, and maintains suspension stability [149]. In conjunction with the above article, it was found that researchers are beginning to look at the effect of mixed nanoparticles on the stability of nanofluids. The molecular force between the mixed nanoparticles can prevent the nanoparticles from agglomerating and achieve high stability. Mixing multiple types of nanoparticles allows the properties of different nanoparticles to be combined for better oil repellent performance. At the same time, the molecular forces between different nanoparticles should be taken into account when mixing nanoparticles of different types, sizes and shapes. Mixed nanofluids with different volume fractions were prepared in a single-step method by Fikri et al., and TiO_2_ and SiO_2_ formed mixed nanoparticles by a volume ratio of 7:3. It was found that the stability of the mixed nano-fluid with a volume fraction of 1.00% was the highest [150].

**Figure 14:**
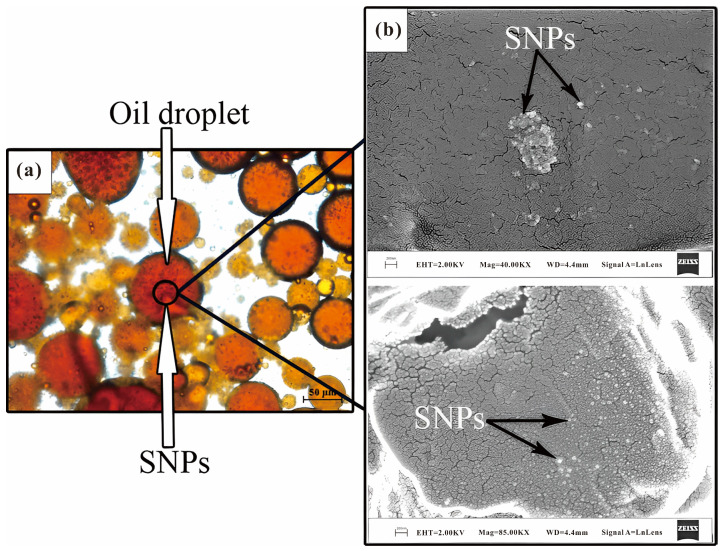
Image of deposition of nanoparticles on oil droplets in a 5000 ppm XG-0.3 wt% SNPs nanoparticle-polymer fluid: (**a**) microscope analysis (**b**) FESEM analysis [6].

Based on the systematic summary of the literature on improving fluid stability of nanoparticle-polymers, the precipitation method and Zeta potential measurement method are analyzed in this paper. Nanoparticles tend to form clusters and aggregates over time due to their high van der Waals gravitational attraction. As the most intuitive method to evaluate the stability of nanofluids, the precipitation method has been widely used by many researchers. The main method is to record the deposition process of the nanoparticles through photographs and observe the change in the weight or volume of the sediment over time [151]. Over time, the larger agglomerated nanoparticles in the solution showed a precipitation behavior, while the smaller agglomerated nanoparticles remained in a suspended state. It was found that the addition of nanoparticles and polymers was beneficial to the stability of the displacement solution by observing the stability of the nano-system based on Xanthan gum and water. Although nanoparticles are conducive to the development of stability, small-scale precipitation still occurs. Figure 14 shows the precipitation of nanoparticles in 5000 ppmXG-0.3 wt% SNPs system [6]. A similar phenomenon was reported by Sharma et al., who used a solution of PAM with a concentration of 1000 ppm as a dispersant and the stability of different SiO_2_ concentrations (0.5 to 2.0 wt%) was visually observed. At the same time, the particle size distribution of nanoparticles was measured by the DLS method, and the experimental results show that the precipitation behavior is related to polymer concentration, nanoparticle size and time.

**Figure 15:**
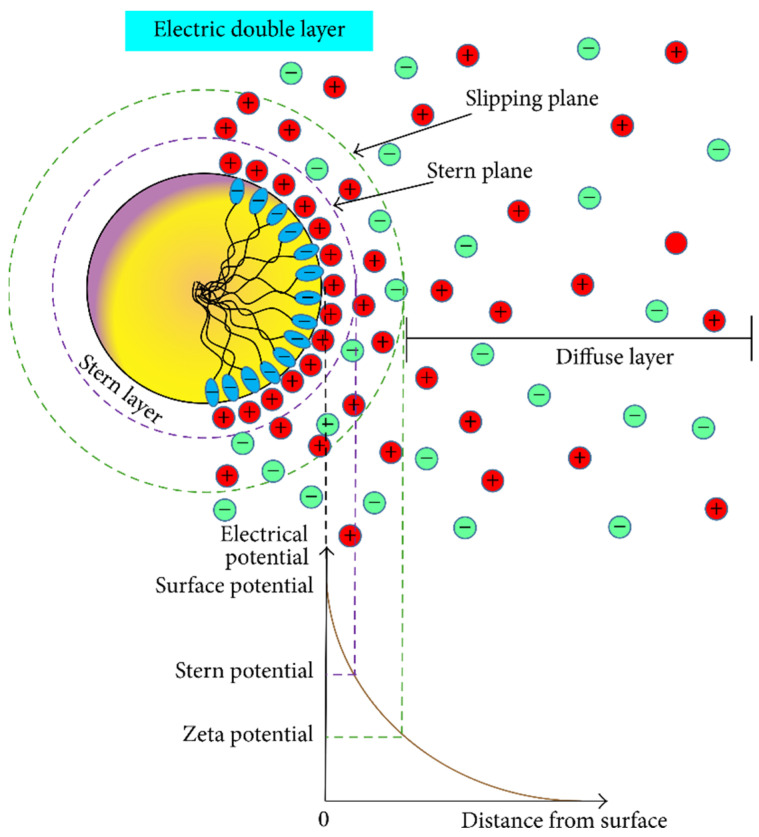
Schematic diagram of double layer repulsive force [154].

Zeta potential measurement is often used to evaluate the stability of suspensions. The Zeta potential, consisting of electrostatic repulsion force and van der Waals force, is a comprehensive reflection index [152]. Electrostatic repulsion exists between adjacent nanoparticles. Because the charge on the surface of the nanoparticles is the same, which inhibits the coalescence of the particles, the stability of nanofluids is improved. On the contrary, the Van der Waals force is gravity that has the opposite effect of electrostatic repulsion, which is detrimental to the stability of nanofluids [153]. As shown in Figure 15, ions with opposite charges are distributed around the nanoparticles. The Zeta potential (repulsive force) formed between the diffusion layers effectively prevents the nanoparticles from contacting and agglomerating [154]. Therefore, the higher the potential value of Zeta, the greater the repulsion force, and the better the stability of the nanofluid.

The Zeta potential measurement method has been widely used in experiments. The Zate potential values for two systems of polymer-nanoparticles and polymer-surfactant -nanoparticles under the same experimental conditions were compared by Sharma et al. The potential of polymer-nanoparticle system was −53.1 mV on day 1, but decreased slightly to −51.2 mV and −49 mV on day 7 and day 27, respectively, corresponding to potentials of −47.1 mV, −45.2 mV and 41.3 mV for the polymer-surfactant-nanoparticle system. The results show that the potential energy of the two systems decreases with the passage of time, but the stability of both systems can be guaranteed, and the stability of the polymer-nanoparticle system is higher than that of the latter [97]. Particle size and zeta potential were measured by the dynamic light scattering method. The potential change of the nanoparticle-polymer fluid with the same concentration does not exceed 4 mv in 10 days, which indicates that xanthan gum nanofluid can still maintain superior stability at 30 °C [6]. The potential of a polymer-citrate coated Fe_3_O_4_ solution was measured at elevated temperature by Izadi et al. The results showed that the solution was very stable in water except at 500 ppm, and the potential change of the solution did not exceed 4 mv at a temperature of 85 °C [4]. Nanoparticle-polymer fluid can maintain a high potential as seen in the above example. The higher the absolute value of the Zeta potential, the smaller the size of the nanoparticles in the fluid. Therefore, stable dispersion can be formed among nanoparticles in the fluid under high repulsive forces.

## 6. Current Challenges

The introduction of nanoparticles into polymers inhibits the hydrolysis and degradation of polymers and forms a network structure between molecules, which reduces the oil-water interfacial tension, increases the viscosity of the system, improves the pseudoplastic behavior of the polymer solution and enhances the stability of the system. The viscosity-increasing property, temperature resistance, salt resistance and anti-shear property of the nanoparticle-polymer fluid are improved in different degrees compared with the traditional polymer. A nanoparticle-polymer fluid can expand the sweep volume, improve oil displacement efficiency, reduce remaining-oil saturation and improve oil recovery. Nano polymer fluid has better reservoir applicability and has a good prospect for application in high temperatures and high salt reservoirs. However, one of the key remaining challenges is that the mechanism and dynamics of oil displacement by nanoparticle-polymers needs to be further investigated in order to provide comprehensive insight into the reservoir-suitability principles of nanoparticle-polymers.

There are many nanoparticles in nano polymer fluid, and their dispersion degree is affected by temperature, salinity, PH, polymer properties, clay content and other factors. Nanoparticles tend to aggregate and form large-size clusters under complex reservoir conditions, which causes the grain size of the particle to increase greatly and eventually lose its unique properties. In addition, pore permeability and pore-throat structure are destroyed by the precipitation behavior of nanoparticles. Therefore, it is necessary to consider the pressure drop caused by permeation, as well as various important factors such as transport behavior and the surface-deposition tendency of the nanoparticle. At present, the lack of basic research on the dispersion degree of nanoparticles in polymer solutions and their transport behavior in reservoir pores is a challenge for the petroleum industry.

Nanoparticles can improve the stability of polymers in complex stratigraphic environments to a certain extent through the findings of the above researchers. However, temperature and salinity still have a certain influence on the properties of polymer fluid nanoparticles. Therefore, the stability of nanoparticle-polymer fluid is also an urgent challenge to be solved in the future.

Large quantities of nanoparticles are injected into the polymer solution throughout the oil extraction operation. It is necessary to reduce the production cost of nanofluids as the price of oil falls and chemical product prices rise. This is also the economic premise for the wide application of nanoparticle-polymer fluid from the laboratory to the field. The sustainable utilization, non-toxicity and innocuous nature of nanoparticles and polymers should also be considered. The actual formation environment and abnormal properties of each oil well have a certain impact on the practical application of nanoparticle-polymer fluids. This has led to the study of nanoparticle-polymer fluids being limited to the laboratory rather than practical applications in the field.

## 7. Future Prospects

This study will help field personnel to select suitable nanoparticles and polymers and increase the feasibility and practical significance of nanoparticle-polymer fluids in practical applications. Two combination methods of polymer-coated nanoparticles and polymer nanoparticles can be selected according to the actual situation of reservoir conditions. These options can effectively overcome the aggregation and agglomeration of nanoparticles in the reservoir environment and increase the displacement efficiency of the nanoparticle-polymer fluid.

With the increasing demand for oil and the urgent need to extract residual oil, nanoparticle-polymer fluid flooding technology has the potential to replace the traditional chemical flooding technology in the future due to its numerous advantages. It is found that SiO_2_ and Al_2_O_3_ have a higher recovery rate than other nanoparticles through the above description and silica has been widely used in tertiary oil recovery due to it having the characteristics of low cost, high purity, a large specific surface area, convenient surface modification and being environmentally friendly. Based on the existing research, the study of modified silica and modified polymers is one of the key development directions of nanoparticle-polymer fluids in the future.

At present, most research on polymeric nanofluids is limited to the study of individual nanoparticles, while the research into hybrid nanoparticles has only been reported in a few papers. Therefore, the synergistic effect of various types of nanoparticles in polymer solutions is a future research directions. In addition, synthetic nanoparticles and polymers can be harmful to the environment, which requires us to further explore ecological issues and strengthen the exploration and research of nanoparticle-polymer fluid blends. Therefore, the recovery of nanoparticles needs further research, and more attention should be paid to the preparation of green nanoparticle-polymer fluid from natural materials.

Supercritical CO_2_ has been widely used in the petroleum field because CO_2_ is non-polluting, non-combustible, cheap and easy to obtain. This property provides the possibility for the collaborative displacement of residual oil by supercritical carbon dioxide and nanoparticle-polymer fluid. Therefore, experiments on the combination of supercritical carbon dioxide with nanoparticle-polymer fluid should be attempted.

## 8. Conclusions

Nanoparticle-polymer fluids have brought revolutionary changes to the development of the oil industry. This paper introduces the polymers commonly used in enhanced oil recovery, and the influence on polymer properties of the external environment such as salinity, high value cations, pH valency and temperature as well as the polymer’s own structure were introduced. Currently, commonly used nanoparticles include non-metallic oxides and metal oxides. The unique properties of nanoparticles are used to improve the performance defects of polymers under harsh conditions. The addition of nanoparticles reduces the degradation degree of polymers, prevents the linear shrinkage of the flexible connection of the polymers, reduces the repulsive force between ions, and increases the viscosity of polymer solutions.

Nanoparticle-polymer fluids exhibit properties that the polymers cannot exhibit alone by introducing nanoparticles into the polymer solution. The viscosity of the displacement solution, oil-water interfacial tension, rock wettability and other properties are improved through the interaction between nanoparticles and polymers, which has a more obvious positive effect on enhanced oil recovery. The research of this paper has a more practical significance to the practical application of the field situation. However, the stability of nanoparticle-polymer fluids needs to be further investigated when operating under complex formation conditions. On the other hand, the study of the oil displacement system with supercritical carbon dioxide incorporated into nanoparticle-polymer fluid is the focus of enhanced oil recovery in the petroleum industry.

## Figures and Tables

**Table 1:** Comparison of IFT, contact angle and oil recovery between polymer and nanoparticle-polymer.

Nanoparticle and Concentration (wt%)	Polymer and Concentration (ppm)	Porous Media	IFT (mN/m)		Contact Angle (°)		Oil Recovery (%OOIP)		Ref.
			P	With NPs	P	With NPs	P	NPF	
SiO_2_ 0.5	HPAM 800	Sandstone	-	-	-	-	26.32	35.0	[96]
SiO_2_ 0.1–0.5	PAM 1000	Glass micromodel	-	-	-	-	50.6	61.8	[72]
SiO_2_ 0.1–0.5	PAM 2000	Glass micromodel	-	-	-	-	55.0	65.0	[72]
SiO_2_ 0.1–0.5	PAM 3000	Glass micromodel	-	-	-	-	58.0	68.0	[72]
SiO_2_ 1.0	PAM 1000	Sandstone	18.03	10.22	-	-	58.1	62.2	[97]
SiO_2_ 0.1	Xanthan gum 5000	Sandstone	17.8	10.75	72.7	22.8	14.5	16.3	[6]
SiO_2_ 0.3	Xanthan gum 5000	Sandstone	17.8	8.67	66.5	-	-	-	[6]
SiO_2_ 0.5	xanthan gum 5000	Sandstone	17.8	8.54	50.4	18.84	14.5	20.8	[6]
SiO_2_ 0.1	HPAM 2000	Sandstone	21.8	11.5	100.3	78.6	54.7	60.81	[98]
Al_2_O_3_ 0.1	HPAM 2000	Sandstone	21.8	9.3	100.3	60.6	54.7	65.3	[98]
Surface Modified Clay	HPAM 2000	carbonate	-	-	-	-	25.0	34.0	[99]
SiO_2_ 0.2	Guar gum 4000	Sandstone	-	-	-	115.0	23.02	35.97	[100]

**Table 2:** The types of NPS and polymers used for EOR and the corresponding recovery efficiency.

Nanoparticle	Polymer	Preparation Method	Contact Angle (°)		IFT (mN/m)		Rock	Recovery Factor OOIP (%)	Ref.
			Water	NPF	Water	NPF			
Bare silica nanoparticles	PDMAEMA homopolymer	Surface grafted	-	-	39.5	19.0	-	-	[119]
SiO_2_	DMAEMA polymer	Coated	75.9	62.2	27.0	14.0	Sandstone	9.9	[120]
Fe_3_O_4_	Anionic polymer	Coated	160.0	114.0	11.23	7.92	Carbonate rock	16.2–17.1	[4]
SiO_2_/Al_2_O_3_	Methacrylate	Coated	143.3	48.75	10.3	6.5	Sandstone	6.0	[121]
Surface modified SiO_2_ (AANP)	Xanthan gum	Radical polymerization method	-	-	31.5	25.1	-	18.5	[122]
TiO_2_	Xanthan gum	Homogeneous miscible	148.0	28.0	24.5	10.8	Natural rock	25.0	[123]
ZnO/SiO_2_	Xanthan gum	Coated	132.6	34.1	19.68	9.45	Carbonate rock	19.3	[1]
SiO_2_	Guar gum	Homogeneous miscible	115.0	73.0	-	-	Sandstone	44.28	[100]
Nano	Cellulose	Surface grafted	101.8	56.8	-	-	Carbonate rock	6.0	[124]
Janus GO	Cellulose nanocrystals	Radical polymerization method	166.0	37.0	20.2	14.4	Edwards White	22.96	[125]
CuO/TiO_2_	PAM	-	151.0	14.7	28.0	15.0	Carbonate rock	-	[2]
ZnO	PAM	Homogeneous miscible	145.86	83.6	29.18	5.52	Carbonate rock	26.34	[126]
Aminated SiO_2_ (M-SiO_2_)	PAM	Radical polymerization method	-	-	-	-	-	17.5	[127]
Surface modified SiO_2_ (C-SiO_2_)	PAM/PEI	Radical polymerization method	-	-	-	-	-	-	[128]
Al_2_O_3_	HPAM	Radical polymerization method	111.8	25.1	-	-	Sandstone	37.6	[5]
SiO_2_	HPAM	Radical polymerization method	111.8	31.0	-	-	Sandstone	33.0	[5]
aminated SiO_2_	HPAM	Homogeneous miscible	-	-	24.0	14.0	-	17.3	[116]
Dispersible nano-SiO_2_	HPAM	Homogeneous miscible	141.8	-	-	-	Quartz sand	10.54	[129]
Aminated GO	HPAM	Radical polymerization method	-	-	-	-	-	-	[130]
Bisfunctionalized GO	HPAM	Radical polymerization method	-	-	-	-	-	21.5	[131]

**Table 3:** The hydrophilic range and chemical bond properties of nanoparticles.

Particle Wettability	Contact Angle Range (°)	Characteristics of Surface Unsaturated Bonds
Strong hydrophilic particles	0	Metal bond, ionic bond
Weakly hydrophilic particles	0–40	Surface ionic bond or covalent bond
Hydrophobic particle	40–90	Molecular bonds are dominant, and local regions are strong bonds
Strongly hydrophobic particles	>90	Intermolecular bonding force

## Data Availability

Not applicable.
